# The Sequential Direct and Indirect Effects of Mountain Uplift, Climatic Niche, and Floral Trait Evolution on Diversification Dynamics in an Andean Plant Clade

**DOI:** 10.1093/sysbio/syae011

**Published:** 2024-03-30

**Authors:** Agnes S Dellinger, Laura Lagomarsino, Fabián Michelangeli, Stefan Dullinger, Stacey D Smith

**Affiliations:** Department of Botany and Biodiversity Research, University of Vienna, Rennweg 14, 1030 Vienna, Austria; Ecology and Evolutionary Biology, University of Colorado Boulder, 1800 Colorado Ave., Boulder, CO 80309-0334, USA; Shirley C. Tucker Herbarium, Louisiana State University, 202 Life Sciences Building, Baton Rouge, LA 70803, USA; Institute of Systematic Botany, The New York Botanical Garden, 2900 Southern Blvd, Bronx, NY 10458, USA; Department of Botany and Biodiversity Research, University of Vienna, Rennweg 14, 1030 Vienna, Austria; Ecology and Evolutionary Biology, University of Colorado Boulder, 1800 Colorado Ave., Boulder, CO 80309-0334, USA

**Keywords:** Adaptive radiation, disparity through time, diversification rates, ecological opportunity, historic biogeography, mountain uplift, paleoenvironment, phylogenetic path analysis, trait evolution

## Abstract

Why and how organismal lineages radiate is commonly studied through either assessing abiotic factors (biogeography, geomorphological processes, and climate) or biotic factors (traits and interactions). Despite increasing awareness that both abiotic and biotic processes may have important joint effects on diversification dynamics, few attempts have been made to quantify the relative importance and timing of these factors, and their potentially interlinked direct and indirect effects, on lineage diversification. We here combine assessments of historical biogeography, geomorphology, climatic niche, vegetative, and floral trait evolution to test whether these factors jointly, or in isolation, explain diversification dynamics of a Neotropical plant clade (Merianieae, Melastomataceae). After estimating ancestral areas and the changes in niche and trait disparity over time, we employ Phylogenetic Path Analyses as a synthesis tool to test eleven hypotheses on the individual direct and indirect effects of these factors on diversification rates. We find strongest support for interlinked effects of colonization of the uplifting Andes during the mid-Miocene and rapid abiotic climatic niche evolution in explaining a burst in diversification rate in Merianieae. Within Andean habitats, later increases in floral disparity allowed for the exploitation of wider pollination niches (i.e., shifts from bee to vertebrate pollinators), but did not affect diversification rates. Our approach of including both vegetative and floral trait evolution, rare in assessments of plant diversification in general, highlights that the evolution of woody habit and larger flowers preceded the colonization of the Andes, but was likely critical in enabling the rapid radiation in montane environments. Overall, and in concert with the idea that ecological opportunity is a key element of evolutionary radiations, our results suggest that a combination of rapid niche evolution and trait shifts was critical for the exploitation of newly available niche space in the Andes in the mid-Miocene. Further, our results emphasize the importance of incorporating both abiotic and biotic factors into the same analytical framework if we aim to quantify the relative and interlinked effects of these processes on diversification.

Why and how lineages radiate (i.e., undergo a rapid increase in species diversity) although others do not, remains a major open question in evolutionary biology (Simões et al. 2016; Sauquet and Magallón 2018). Ecological opportunity is commonly regarded as the key element of adaptive radiations (a special case of evolutionary radiations, Wellborn and Langerhans 2015; Simões et al. 2016), with ecological opportunities defined as the availability of ecologically accessible resources that may be evolutionarily exploited (sensu Simpson 1953; Stroud and Losos 2016). Such resources may only be available over short periods of time or in certain areas, that is, due to changing geomorphological or climatic conditions (mountain or island uplift, flooding, glaciation). Adaptive radiations thus require access to these resources, that is, through the colonization of new areas, the absence of ecologically similar (hence competing) species, and high evolvability (i.e., through key innovations) allowing for increased diversification in niche and trait space (called “disparification” from here onwards, Simpson 1953; Schluter 2000; Knouft et al. 2006; Evans et al. 2009; Kozak & Wiens 2010; Burbrink et al. 2012; Stroud & Losos 2016; Alexandre et al. 2017; Kennedy et al. 2018). Importantly, assessments of diversification dynamics across clades in the same area have shown that ecological opportunities do not consistently generate radiations, thus pointing towards the critical roles of timing and a clade’s evolvability in exploiting ecological opportunities (Burbrnik et al. 2012; Stroud and Losos 2016; Valente et al. 2019; Jimenez-Ortega 2023). Further, evolutionary radiations may occur without major shifts in niche or trait space, and hence without increases in niche or trait disparity (i.e., low disparification through niche conservatism, Aguilée et al. 2013; Folk et al. 2019; Hiller et al. 2019). Thus, determining when and how ecological opportunities generate bursts in diversification and disparification is essential for better understanding the complex processes generating biodiversity (Simões et al. 2016; Harmon et al. 2019; Rull 2020).

Traditionally, the individual factors influencing diversification have been studied through two major models: the Court Jester model, postulating a paramount role of extrinsic abiotic factors (geomorphology, biogeography, and abiotic climatic niche evolution), and the Red Queen model, proposing biotic factors (intrinsic traits, species interactions) as main drivers of diversification (Barnosky 2001; Benton 2009). Although substantial support has been found for both models (Court Jester: i.e., Kong et al. 2022; McCullough et al. 2022; Red Queen: i.e., Quental and Marshall 2013; Fernández-Mazuecos et al. 2019; García-Girón et al. 2020; Fraser et al. 2021; Pérez-Escobar et al. 2022), both approaches fall short in that they attribute strong individual effects to the respective a-/biotic factor under study, without exploring the potential for multiple interacting and potentially temporally staggered effects on diversification dynamics (Fig. 1, Wiens 2011; Aguilée et al. 2018; Uyeda et al. 2018; Helmstetter et al. 2023). Testing for the relative contribution of these factors is challenging because a thorough analysis requires the availability of various data types such as a well-sampled, time-calibrated molecular phylogeny, occurrence information, abiotic climatic data, and trait/natural history information (Supplementary Fig. S1), which are difficult to collect for large, widespread clades occurring in remote areas. Moreover, despite rapid developments in phylogenetic comparative methods, unified modeling frameworks allowing for explicit tests of interrelated a-/biotic factors, and their potentially indirect effects on diversification remain scarce (Jablonski 2008; Condamine et al. 2018a; Uyeda et al. 2018).

**Figure 1.**
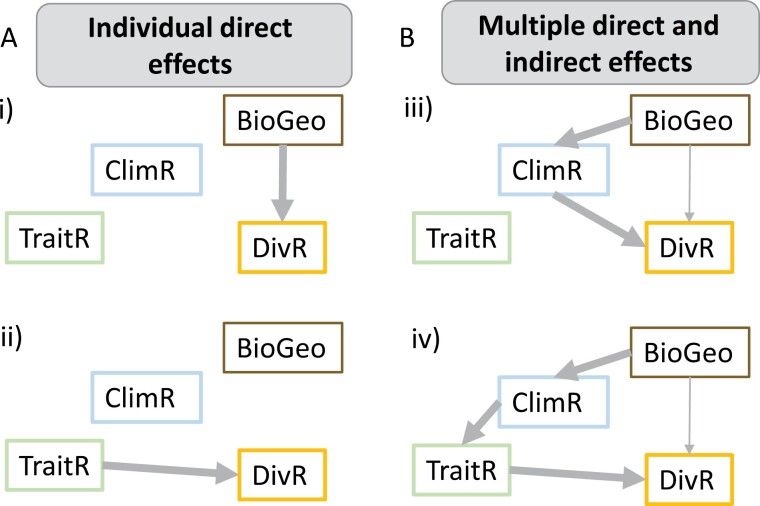
Example graphical models of individual direct or multiple direct and indirect effects of abiotic and/or biotic factors on diversification dynamics. Arrows denote effects, with the width indicating the magnitude. a) Simplest path analytical models might support i) strong effects of biogeographic region (BioGeo) or ii) trait evolution (TraitR) on diversification rates (DivR). b) Possible interactions among these factors and their potentially interlinked, temporally staggered direct and indirect effects on diversification, with two multifactor scenarios where iii) occurrence in a different biogeographic area alters climatic niche evolution (ClimR, i.e., rapid adaptation to new environments), which then affects diversification rates, or iv) occurrence in a different biogeographic area directly impacts rates of climatic niche evolution, which is followed by increased trait evolution (i.e., to adapt to changed biotic interactions in new environments) and then affects diversification rates and results in an adaptive radiation*.*

Graph analytical approaches such as phylogenetic path analyses, although rarely employed, represent a promising way forward as a single modeling framework for testing multiple and potentially interacting abiotic and biotic factors that could influence diversification (proposed by Höhna et al. 2014; Uyeda et al. 2018). By examining the possible drivers alone and in combination, we can compare multiple scenarios modeling the reciprocal (indirect) and direct effects of each. For example, movements between biogeographic regions alone could lead to elevated diversification, for example, under a niche conservatism hypothesis (Fig. 1ai). Alternately, trait evolution (i.e., key innovations) may increase diversification rates (Fig. 1aii). More realistically, these factors interact such that dispersal to a new region leads to shifts and disparification in climatic niche space, which could in turn lead to reproductive isolation and increase diversification (Fig. 1biii). Climatic niche disparification could further lead to increased trait evolution, which ultimately allows for adaptive radiation (Fig. 1biv). Being based on well-grounded biological hypotheses, graphical models hence allow for evaluating the likelihood of different causal linkages between a-/biotic factors (von Hardenberg and Gonzalez-Voyer 2013; Uyeda et al. 2018) and allow us to disentangle their interactions with each other and with diversification dynamics (Lagomarsino et al. 2016; Naujokaitis-Lewis and Fortin 2016; Seeholzer et al. 2017; Aguilée et al. 2018; Barrabé et al. 2019; Quintero and Landis 2019; Vasconcelos et al. 2021; Tribble et al. 2023).

Here, we outline such an integrative approach to studying the relative importance of historical biogeography, geomorphology, timing, and climatic niche and trait evolution on diversification using a neotropical plant clade (tribe Merianieae, Melastomataceae). Merianieae comprise ca. 300 species (46% sampled here) across the Neotropics, and present remarkable variation along abiotic and biotic niche axes. They occupy lowland rainforests to high-elevation grasslands and originated in the Miocene (ca. 30 mya; Michelangeli et al. 2022). Their distribution range encompasses geomorphologically distinct areas, with geologically old (Precambrian) mountains (Guiana shield, Souza et al. 2020; Southern Andes, parts of the Atlantic Forest, Vasconcelos et al. 2020; Guedes et al. 2021; Bacci et al. 2022; Larocca et al. 2022) and recently (mid-Miocene) uplifted mountain ranges (Central America, Northern Andes, Boschmann and Condamine 2022), as well as periodically or formerly submerged areas (Mio-Pliocene marine incursions and changes in drainage systems in lowland Amazonia, Hoorn et al. 2022; gradual emergence of Antillean islands and closure of the isthmus of Panamá, Cacho and Baum 2012, Tejedor and Calatayud 2022; for details see Supplementary Methods). Despite this geomorphological mosaic of ecological opportunities across neotropical areas, the uplift of the Andes is commonly detected as major recent driver of neotropical biodiversity (Gentry 1982; Antonelli et al. 2015; Antonelli 2021; Palma-Silva et al. 2022; Pérez-Escobar et al. 2022), given temporal correlations of North Andean uplift during the Mio-Pliocene and radiations of animals (i.e., lizards: Esquerré et al. 2019; amphibians: Santos et al. 2010; moths: Strutzenberger and Fiedler 2011, Li et al. 2022) and plants (Lagomarsino et al. 2016; Testo et al. 2019; Meseguer et al. 2022; Vieu et al. 2022). Thus, the question arises whether these North Andean radiations were spurred through colonization by species from the older mountain ranges, preadapted to the newly forming Andean environments (Rull 2011; Ledo and Colli 2017; Tejedor and Calatayud 2022) or through de novo abiotic climatic niche and trait evolution upon North Andean colonization.

Given their occupation of such diverse bioregions across the Neotropics, we predict that Merianieae have undergone shifts in abiotic climatic niches and biotic traits potentially impacting diversification dynamics. Shifts in vegetative and physiological traits, for example, have occurred in plant clades globally during adaptation to montane environments (i.e., cushion growth form, reduced leaf area, and perennial habit), and have been interpreted as key innovations for mountain radiations (Simões et al. 2016; Ebersbach et al. 2017; Quintero & Jetz, 2018, Homeier et al. 2021). Shifts in biotic interactions, such as pollination and seed dispersal, are also common across elevational gradients but have received much less attention (Lefebvre et al. 2018; McCabe and Cobb 2021; Dellinger et al. 2023). Although there is increasing evidence for large-scale associations between the abiotic environment and pollination strategies (i.e., insect pollination dominating in warm lowland rainforests, vertebrate pollination dominating in cool, rainy montane forests in the neotropics, Dellinger et al. 2023), it remains unclear whether shifts in biotic interactions are equally important as shifts in vegetative/physiological traits, and whether they represent a prerequisite for colonization of and diversification in mountains or a consequence thereof. With their diversity in vegetative traits (small herbs to large trees) and biotic interactions (pollination by bees, passerines, or mixed assemblages of hummingbirds, bats, or rodents; Dellinger et al. 2014, 2019a, 2021; Michelangeli et al. 2022), Merianieae represent an ideal model for testing hypotheses about the individual or multiple effects of abiotic and biotic evolution on diversification (Fig. 1). To explore these hypotheses, we first reconstruct the biogeographic history of the clade and estimate diversification rate variation across the tree. We next incorporate analyses of climatic niche variation as well as vegetative and floral trait space to test whether disparification is tied to diversification and whether abiotic and biotic disparification coincides temporally. Finally, by synthesizing results from our biogeographic, climatic, and trait evolution analyses into a single path analytical framework (Supplementary Fig. S1), our findings uncover strong links between the colonization of the Andes, niche evolution, and rapid speciation.

## Methods

### Existing Phylogenetic and Floral Trait Data for Merianieae

We used the molecular phylogenetic hypothesis from a family-wide Melastomataceae phylogeny based on two nuclear ribosomal spacers (*ETS. ITS*) and seven plastid regions (*accD-psaI, atpF-atpH, ndhF, psbK-psbI, rbcL, rpl16* and *trnS-trnG*) presented by Reginato et al. 2022 and pruned to 139 tips for Merianieae (46% of species). We delineate seven major clades for our analyses, following the most recent systematic treatment of the tribe by Michelangeli et al. 2022, with the following sampling fractions: *Macrocentrum* 2&3 44%, *Macrocentrum* 1 44%, *Adelobotrys* and *Adelbertia* 43.6%, *Graffenrieda* 44.2%, *Salpinga* 40%, and core Merianieae 48.3%. These major clades are also recognized in a previous study on Merianieae flower trait evolution (Dellinger et al. 2019b, 2021). Backbone relationships between some clades (e.g., *Macrocentrum*, Michelangeli et al. 2022) vary between these two available phylogenies, but, central to our study, core Merianieae consistently form a monophyletic group originating in the mid-Miocene. In the main text, we present results on the phylogenetic hypothesis of Reginato et al. 2022, which has been dated in BEAST v.2.6.3 on a consensus tree for Melastomataceae with constrained topology, using four different combinations of time prior constraints of one secondary calibration point at the stem of Melastomataceae (Silvestro et al. 2021) and three fossils (Duarte 1956; Collinson and Pingen 1992; Carvalho et al. 2021). The phylogenetic hypothesis of Dellinger et al. (2019b) is based on a slightly different taxon sampling (141 spp.) and lacks the fossil of Carvalho et al. (2021), but mean crown ages are comparable across both phylogenies, with the 95% highest probability density ranging from 37.94 to 23.5 Ma (30 Ma mean crown age in Reginato et al. (2022), 24.5 Ma in Dellinger et al. (2019b)). To ascertain robustness of our results, we ran analyses on both phylogenetic hypotheses, and additionally randomly subsampled the phylogenetic hypothesis of Reginato et al. 2022 to only 50% of taxa to evaluate potential bias due to incomplete sampling (Supplementary Table S1 for sampling fractions).

Given our extensive work on pollination and floral evolution in Merianieae (Dellinger et al. 2014, 2019b, 2021), we compiled data on 15 pollination-relevant floral traits (i.e., corolla shape, stamen structure, and appendage types) from Dellinger et al. (2021). We used the pollination syndrome classification from Dellinger et al. (2021); built through random forest analyses, where models were first trained and tested with high prediction accuracy (96.4%) on 32 species with documented pollinators (spread across Merianieae) and then used to classify species with unobserved pollinators (107 in our dataset), Supplementary Fig. S2 and Supplementary Notes S1).

### Historical Biogeographic Analyses

To test how movements among geographical regions influenced Merianieae diversification, we delineated nine areas based on their distinct geomorphological histories (Supplementary Fig. S3, Sánchez-Herrera et al. 2020; Boschman and Condamine 2022; Hoorn et al. 2022): Central America and Mexico, Tumbes-Chocó-Magdalena, North Western Andes, North Eastern Andes, Central Andes, Amazon Basin, Guiana Shield, Atlantic Forest, and Antilles. This delimitation captures geologically “old” areas present when Merianieae originated ca. 30 mya (Central Andes, Guiana Shield, Amazon Basin (including the Cerrado/Caatinga, which only contained two species, Supplementary Table S2), parts of Central America and Atlantic Forest) and comparatively “young” areas (North Western Andes, North Eastern Andes, Tumbes-Chocó-Magdalena area, Antilles) that formed during the diversification history of Merianieae. We classified all species into these areas based on the pruned GBIF occurrence maps; the maximum number of areas occupied was five (two species).

We then used the R package BioGeoBEARS to estimate ancestral range probabilities for Merianieae (Matzke 2013). We focused on DEC (dispersal-extinction cladogenesis, Ree 2005) models because our main aim was to reconstruct ancestral ranges (Ree and Sanmartín 2018). We also ran DEC with time-stratified dispersal multipliers to account for the varying availability and formation of different areas (Dupin et al. 2017). To this end, we constructed dispersal multiplier matrices for four geologically important time strata (TS) in the diversification history of Merianieae: 34–20 Ma, 20–15 Ma, 15–5 Ma, and 5 Ma to present. TS start at 34 Ma, which is before the origin of Merianieae, with the Central Andes mostly uplifted, the North Western Andes uplifted to ca. 50%, and inland lake systems present along the eastern Andean foothills (Boschmann and Condamine 2022). The breaks at 20 Ma and 15 Ma reflect another pulse of uplift of the North Western Andes (Boschmann and Condamine 2022), gradual formation of the Antilles and Central American islands (Crews and Esposito 2020), as well as changes in the Amazonian Pebas lake systems (i.e., potentially hindering dispersal between the Amazon basin and the Andes). Finally, the break at 5 Ma reflects the rapid uplift of the North Eastern Andes, the formation of the formerly submerged Tumbes-Chocó-Magdalena area, with a definite land bridge between Central America and South America (O’Dea et al. 2016). We chose values for dispersal multipliers to range between 1 (dispersal highly possible) and 0.00001 (dispersal highly unlikely; Supplementary Fig. S4). To buffer potential spurious effects of multiplier choice, we repeated a model run including the w parameter, which modifies the dispersal matrices during the maximum-likelihood search by elevating the dispersal multiplier to the power of *w* (0–3, Dupin et al. 2017). In order to explore how the changeable historic connectedness among areas affects ancestral range estimates, we complemented our analyses with a time-stratified model including an adjacency matrix, only allowing for contingent areas (i.e., not separated by marine incursions) at each point in time as ancestral ranges. Finally, to assure comparability of our results to classical biogeographical studies primarily focusing on ecoregion patterns, we also reran our analyses using the 10 biogeographic dominions/zones of Morrone et al. 2022 and compare results in the SI (Supplementary Note S1, Supplementary Figs. S3, S5–S8).

### Diversification Rate Inference

To estimate diversification rate dynamics through time, we used BAMM v.2.5.0 (Rabosky 2014). We estimated initial priors for lambda and mu in BAMMtools v. 2.1.9 (Rabosky et al. 2014), used clade-specific sampling fractions (Supplementary Table S1), and kept the expected number of shifts at 1 (recommended for trees < 500 tips). To assess whether this shift constraint impacted our results, we also ran models with two and five expected shifts. We ran BAMM for 5 million MCMC generations and confirmed a minimum ESS (effective sample size) of 200 after removing 10% burn-in (number of shifts: 2727, log-likelihood -1948). We determined the 95% of credible set of shift configurations (CSS) and the best-fit shift configuration (maximum a posteriori probability) using Bayes factors. To evaluate the robustness of results obtained on the consensus tree, we reran BAMM on 1) 1% of trees from the posterior distribution with either shortest or longest overall branch lengths (to assess the impact of branch length differences), on 2) 1% of random trees from the posterior distribution (to assess the impact of topological differences), and on 3) 1% of trees randomly subsampled to 50% (summarized in Supplementary Tables S3, S4).

Although BAMM reliably estimates “major” rate shifts (Rabosky et al. 2017; Title and Rabosky 2018), smaller but more frequent rate shifts are likely not detected. We hence used two additional (speciation) rate estimates, the DR statistic (allowing for higher variance among tip rates, Title and Rabosky 2018) and recently developed Bayesian approaches to estimate cladogenetic (branch-specific) shifts in diversification rates (ClaDS, Maliet et al. 2019). We estimated the DR statistic following Jetz et al. (2012). For ClaDS, we estimated priors through a pure-birth model (ClaDS0) and then estimated branch-specific speciation rates using a model allowing for a constant turnover rate through time (ClaDS2, RPANDA v 1.9, Morlon et al. 2016). Recently, critique on the nonidentifiability of diversification rates from phylogenetic data has called most of the classic methods for estimating speciation and extinction rates in question (Louca and Pennell 2020). The ClaDS2 model mitigates some of these risks because it restricts the parameter estimates to well-justified prior assumptions (i.e., rate shifts are unlikely on very short branches; rates are correlated across the tree; extinction rates are variable across the tree although species turnover is constrained; Morlon et al. 2022). We ran ClaDS2 for 10000 MCMC iterations, with a thinning rate of 200 and including a sampling fraction of 0.46. We extracted the maximum a posteriori branch-specific rate estimates for subsequent analyses.

### Historical Geomorphological Processes

In order to test whether major geomorphological events during the Miocene (global cooling, Andean uplift) shaped Merianieae diversification, we used the statistical framework outlined by Condamine et al. 2018b, fitting a series of birth-death models in dependence on historic processes. For historic variables, we retrieved paleotemperature (mirroring major trends in global climate change) and paleoelevation (estimated separately for the Northern Andes) data from Boschman and Condamine 2022. Following Boschman and Condamine 2022, we fit 14 diversification models: two with constant diversification rates (null models for comparison), four with time-dependent diversification rates, four with temperature-dependent diversification rates, and four with elevation-dependent diversification rates. We use functions fit_bd (time-constant and time-varying models) and fit_env (temperature- and elevation-dependent models) from R package RPANDA 1.9 (Morlon et al. 2016), specified a sampling fraction of 0.46 and spline interpolation for the paleo-variables (degrees of freedom 80). We assumed an exponential dependency of speciation (*λ*) and/or extinction (*µ*) rate on time (*t*) or the environmental factors (T—paleotemperature; A—Andean paleoelevation, see Supplementary Notes S1 for details). We fitted each model on the consensus phylogeny, a random subset of 100 trees and the ten trees subsampled to 50% by maximum likelihood, starting with the simplest (constant rate) models. Because optimization algorithms can be sensitive to the choice of initial parameters, we used parameter estimates from simpler models to inform the starting values of more complex models (Boschman and Condamine 2022). We selected the best-fitting model for each type of model using Akaike weights (AICw) and compared the selected best-fit models between model types using corrected Akaike Information Criterion (AICc, Burnham and Anderson 2011).

### Collation of Climatic Niche and Vegetative Trait Data

To derive climatic niche data, we compiled occurrence records from GBIF (accessed July 23, 2021, 24,682 records before filtering), the speciesLink repository as well as from Latin American herbaria through data provided by colleagues (Colombia—Humberto Mendoza, Peru—Robin Hilario, Ecuador—Agnes Dellinger, 7102 records). We submitted these data to standard cleaning procedures (CoordinateCleaner, Zizka et al. 2019), leaving 9134 pruned occurrence records. We plotted these pruned occurrences for each species separately to visually verify whether they correspond to the known distribution ranges and to identify areas lacking geo-referenced locations. For occurrences outside of hitherto documented areas, we checked the respective herbarium voucher (when digitized) to verify correct identification. For species with large gaps in the documented distribution range, or fewer than ten georeferenced records (24 species), we searched the literature and herbarium vouchers for additional localities, and georeferenced these points (see Supplementary Note S1 for details).

As last data validation step, we extracted elevation data for each record (30 m, Aster GDEM v.3, AppEEARS Team 2021; accessed 12/19/2021; 1 km, GMTED2010, Danielson and Gesch 2011) and used boxplots to compare the elevational distribution range retrieved from GBIF records and the elevation model-based datasets for each species, removing outliers (Notes S3). Finally, we thinned occurrences to one occurrence/species/1 km grid cell (*gridSample, dismo*), leaving 5876 occurrences (median of 20 occurrences/species, 20 species with fewer than five occurrences).

We obtained climatic niche variables for each record from 19 bioclimatic layers, the net primary productivity layer, and the mean monthly total cloud cover layer of CHELSA v2.1 (http://chelsa-climate.org/) at 1 km resolution (Karger et al. 2017), and calculated median values for each species.

To obtain vegetative trait data, we scored growth form (tree, shrub, scandent shrub, liana, and herb), leaf margin (entire and toothed), and leaf thickness (membranaceous/chartaceous, subcoriaceous, and coriaceous), and measured leaf area (mm²) from digitized herbarium vouchers (one mature leaf of three specimens/species) using TraitEx 2.0 (Triki et al. 2019). We chose these traits because of their functional relevance (i.e., thicker leaves as protection from cold or increased UV radiation in mountains, Supplementary Note S2 for details).

### Estimating Climatic Niche, Vegetative, and Floral Trait Disparity and Evolution

To assess the relative contribution of climatic, vegetative, and floral niche evolution in Merianieae diversification, we combined multivariate statistics with comparative methods. First, we assessed general aspects of niche disparity using morphospaces. For the climatic dataset (comprised of continuous data only), we calculated a PCA on the scaled and centered environmental data (*prcomp*). For the vegetative and floral trait dataset (different data types), we calculated PCoAs based on Chartier et al. (2017). To visualize patterns of niche space occupation, we calculated phylomorphospaces (*phytools*, Sidlauskas 2008; Revell 2012), extracted scores for each species from the first two (vegetative and climatic) or three (floral) PC axes and tested whether the six Merianieae clades differ in occupation of either space using permutational analyses of variance (perMANOVA, *pairwiseAdonis* with Bonferroni correction for multiple comparisons, Martinez Arbizu 2020) and calculated disparity as the per-clade average squared pairwise distances among coordinates (*dispRity,*Guillerme 2018). Because clades differ in size (4–75), we repeated disparity analyses by rarefying each clade randomly to four species 100 times and tested for significant differences among clades using the Bhattacharyya coefficient (*test.dispRity*, Guillerme 2018).

To quantify the temporal component of niche diversification, we ran disparity-through-time (DTT) analyses on the respective two/three PC axes following Harmon et al. 2003 (*dispRity,*Guillerme 2018; Supplementary Note S1 for details). Using simulations (1000 permutations), we tested whether DTT significantly differed from a random process, and ran analyses across five different time bins (present—30 my,—24 my,—18 my,—12 my,—6 my). Although DTT analyses on incomplete phylogenies (like ours) may overestimate disparity towards the present, we want to highlight that we are using DTT analyses to compare disparification among climatic niches and traits, and these relative patterns are comparable regardless of phylogenetic completeness.

To test whether changes in niche disparity indicate changes in selection regimes, we ran Ornstein-Uhlenbeck models on the three morphospaces. We fit explorative OU models without a priori specification of selection regimes for each axis separately (l1OU, Khabbazian et al 2016), and compared the divergent shift-model with a convergence model using pBIC. Further, we tested three hypotheses on niche evolution (M1—Andean model: a single shift in selection regime coinciding with Andean colonization; M2—core Merianieae model: a single shift in selection regime with core Merianieae; M3—pollinator shift model: seven shifts within core Merianieae; Supplementary Methods, Fig. S9) and compared their fit against the neutral models using pBIC.

### Exploring the Effects of Biogeography, Niche, and Trait Evolution on Diversification

**Figure 2.**
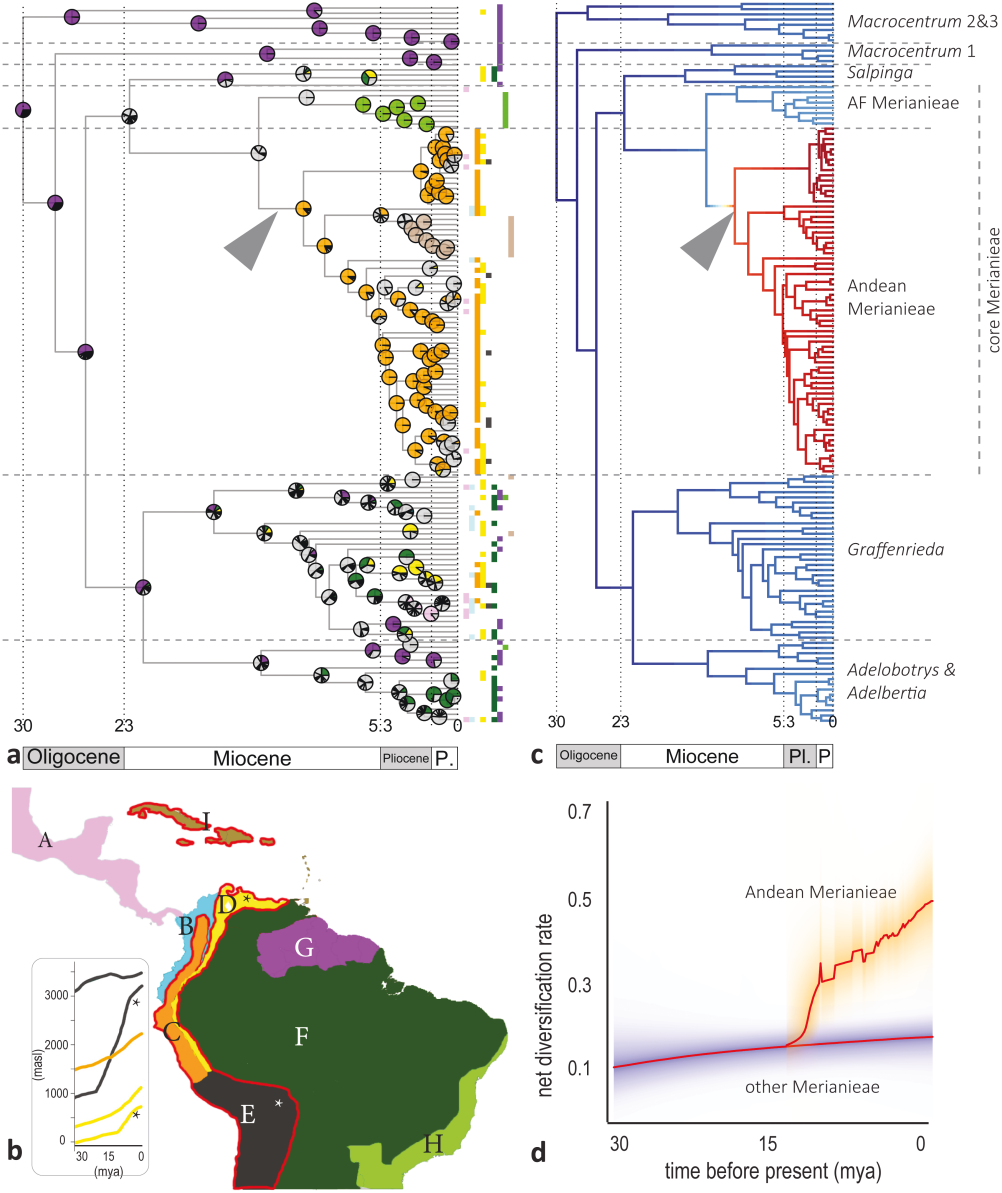
Biogeographical history and diversification dynamics of Merianieae. a) Time-calibrated molecular phylogeny of Merianieae with present-day biogeographical distribution (tips) and reconstruction of historical biogeography (time-stratified DEC + J model), with colored pies showing historical areas; strongly dissected or light gray pies indicate high uncertainty about historic ranges. Colors for areas follow the map in b), arrow indicates colonization of the Western Andes 15-12 mya. b) Map of the Neotropics with geomorphological areas: A) Central America and Mexico, B) Chocó-Tumbes region, C) North Western Andes, D) North Eastern Andes, E) Central Andes, F) Amazon Basin (including Cerrado, Caatinga, and Chaco), G) Guiana Shield mountains, H) Atlantic Forest; red line indicates the range of Andean Merianieae (including the Antillean clade) with increased diversification rates (c); insert on the left indicates history of Andean uplift (adapted from Boschman and Condamine 2022) with colored lines representing C–E, D and E represented by separate uplift estimates for western and eastern (asterisks) cordilleras. c) Bayesian Analyses of Macroevolutionary Mixture (BAMM) show one significant increase in diversification rate in the Andean Merianieae clade (to 0.5), whereas diversification rates only increased very slowly (to 0.16) in all other clades. d) Rate-through-time plot for core Merianieae versus all other Merianieae.

Finally, we used phylogenetic path analyses (Supplementary Fig. S1, von Hardenberg and Gonzalez-Voyer 2013; Gonzalez-Voyer and Von Hardenberg 2014) to synthesize our results and estimate the individual or multiple direct and indirect effects of biogeography, climatic niche and trait evolution on Merianieae diversification. Phylogenetic path analyses are a class of phylogenetic regressions that account for the nonindependence of species by directly incorporating the phylogeny into models of trait evolution through processes such as Pagel’s lambda. We used tip rates to include diversification in these analyses, assuming that present-day tip rates are informative of past evolutionary processes, consistent with the continued differences in diversification and niche/trait evolution rates across Merianieae clades (Fig. 2, Supplementary S10). We estimated tip rates for diversification through BAMM (diversification rate), the DR statistic, and ClaDS (speciation rates). To obtain tip rate estimates for climatic, vegetative, and floral evolution along PC axes 1–3, we used phylogenetic ridge regression (Supplementary Fig. S10, *RRPhylo*, Castiglione et al. 2018). To incorporate a biogeographic background, we binarized the present-day distribution for each species into Andean (including the Central American cordilleras) or extra Andean (including species with wide distribution ranges, but primarily found at lower elevations). We then constructed eleven increasingly complex models of varying dependence of speciation rates (SR) on biogeographic background (BG), rates of climatic niche evolution (climR) and vegetative (vegR) and floral (florR) trait evolution, testing both for individual effects of single factors (e.g., BG or climR alone impacting SR), or sequential direct and indirect effects of multiple factors (e.g., BG impacting climR, and both impacting SR; Fig. 1, Supplementary Figs. S10, S11). The simplest models (one to four; individual effects) included nine independencies, and the most complex model (11; multiple effects) included two independencies (Supplementary Fig. S12). We tested these models through phylogenetic path analyses as implemented in the R package *phylopath* (Van der Bijl 2018), using Pagel’s lambda for the regressions on continuous variables (SR, niche/trait rates), and maximum penalized likelihood for the binary variable (BG; Ho and Ané 2014). To assure model fit, we constrained *λ* between 0 and 1 (less to more influence of shared history). Following Gonzalez-Voyer and Von Hardenberg (2014), we then used Fisher’s *C* statistic to evaluate model fit through the *d*-separation test, with a model fitting the data well having a *P*-value larger than 0.05. We compared fit across models using the corrected *C*-statistic Information Criterion (CICc), with differences in CICc < 2 indicating models with similar support (Cardon et al. 2011; Gonzalez-Voyer and Von Hardenberg 2014). If two models had a delta CICc < 2, we averaged across models. Finally, we calculated relative likelihoods and CICc weights of models to evaluate the relative strength of evidence for each path model given the data and set of models (Burnham et al. 2011).

## Results

### Biogeography, Diversification Dynamics, and Historical Processes in Merianieae

Merianieae are widely distributed across the Neotropics (Fig. 2). *Macrocentrum* 1, 2, and 3 are mostly restricted to the ancient Guiana shield mountains, whereas *Adelobotrys* and *Adelbertia* and *Salpinga* are widely distributed across lowland rainforests in the Amazon Basin, Amazonian foothills, Atlantic Forest, Chocó, and Central America (Fig. 2). The genus *Graffenrieda* is most widespread and has colonized both lowlands and mountains. Core Merianieae show a disjunct distribution, with a small clade found in the Atlantic Forest, and a large clade in the recently uplifted Northern Andes and Central American mountains, with a small clade of Antillean species nested within (Fig. 2).

Across DEC models, the Guiana shield is reconstructed as likely ancestral range for Merianieae, and specifically for clades with present-day distributions in the Guiana shield or Amazon basin (Fig. 2, Supplementary Fig. S5). The nodes leading to core Merianieae and Atlantic Forest Merianieae cannot be reconstructed reliably (Fig. 2a). Importantly, however, across analyses, we see one major colonization event of the North Western Andes ca. 13.7–10 million years ago by Andean Merianieae, with little movement across the Andes (Fig. 2a in orange) and a single dispersal to the Antilles (Fig. 2a in brown) ca. three million years ago. Finally, colonization of the most recently uplifted North Eastern Andes (Fig. 2b) occurred within the past five million years across five of the seven Merianieae clades, but has not resulted in major radiations (Fig. 2a in yellow). These patterns are consistent across the 10 trees randomly subsampled to 50%, a different phylogenetic hypothesis and a biogeographic area delimitation following ecoregions (Supplementary Fig. S6–S8).

Merianieae show 77% probability of a single, 3-fold increase in diversification rates (Fig. 2c, d). The highest probability (89.9%) for this rate shift is along the branch to Andean (and Antillean) Merianieae, second highest probability (7.5%) along the branch to core Merianieae (also including Atlantic Forest Merianieae; Supplementary Fig. S13). We recovered this same single rate shift across the 10 extreme trees (shortest/longest branches), 10 random trees, 10 trees randomly subsampled to 50%, when setting the expected number of shifts to 2 and 5, and when using the phylogenetic hypothesis of Dellinger et al. (2019b) (Supplementary Fig. S14, Supplementary Tables S3, S4). When estimating branch-specific diversification rates through ClaDS, we also found overall higher speciation rates among Andean Merianieae (Supplementary Figs. S15, S16).

Comparing the effect of evolutionary time, climatic cooling, and Andean uplift as drivers of diversification in Merianieae, models with Andean uplift had slightly higher support than other models (AICω = 0.38, *α* 0.003; 73/100 trees; second highest support for time-dependent process, AICω = 0.134, *α* −0076; 25/100 trees). All paleo-elevation models showed a weak positive correlation between speciation rate and Andean elevation (*λ*_0_ = 0.012 events/Myr; faster speciation with higher elevation). The time-dependent models showed negative correlations with speciation rates (*λ*_0_ = 0.402 events/Myr, increase in speciation rates over time). The temperature-dependent model was never selected as best-fit (Supplementary Table S5). Overall, support of the best-fit models was above AICω 0.071, whereas a lower value would be expected if all models were equally likely (1/14 models). These results are supported by analyses of the phylogenetic hypothesis of Dellinger et al. (2019b) (Supplementary Table S5, paleo-elevation model AICω = 0.241; 83/100 trees, time-dependent process AICω = 0.196; 14/100 trees).

### Climatic Niche, Vegetative, and Floral Trait Disparity and Evolution

**Figure 3.**
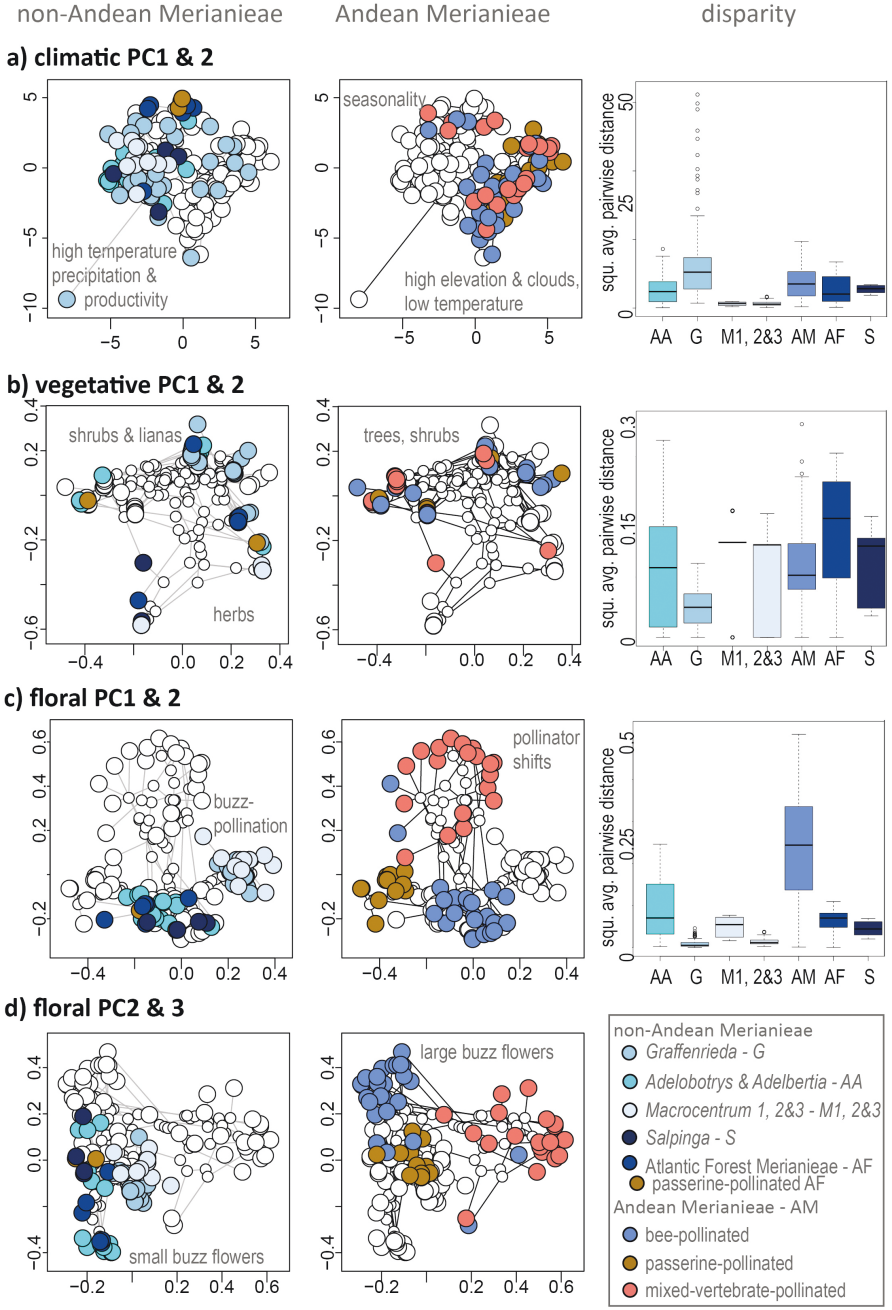
Phylo-niche spaces and niche disparity (rarefied to 4 spp/clade) of Merianieae. a) Climatic niche space (PC axes 1 and 2 (44.7% and 27.7% variance explained)) shows distinct niches for non-Andean and Andean Merianieae, the first associating with higher temperatures, precipitation, and productivity, whereas the latter associated with cooler mountain climate. Atlantic Forest and Antillean Merianieae overlap in niche space characterized by higher seasonality. Graffenrieda showed the highest climatic niche disparity, followed by Andean Merianieae. b) Vegetative trait space (PC axes 1 and 2 (31.8% and 22.9% variance explained)) showing niche overlap of non-Andean and Andean Merianieae, and a separation of smaller-leaved, herby growth forms and larger-leaved shrubs. Vegetative disparity does not mirror climatic disparity, and highest disparity is found in Atlantic Forest Merianieae. c) Floral trait space along PC axes 1 and 2 (19% and 18.5% variance explained) shows distinct niches for vertebrate-pollinated Andean Merianieae, whereas bee-pollinated Andean and non-Andean Merianieae overlap in niche space. Graffenrieda and Macrocentrum, characterized by small (< 1cm) whereas flowers with stamens forming a cone cluster in a separate area of niche space, and show little disparity. Highest disparity is found in Andean Merianieae. d) Floral trait space along PC axes 2 and 3 (16.5% variance explained) shows a shift in niche space of bee-pollinated Andean Merianieae, characterized by large, sturdy, pollen-rewarding flowers with spread-out, colorful stamens buzzed by large montane bees (e.g., Centris, Eulaema, Xylocopa, Bombus) whereas non-Andean Merianieae are visited by smaller bees (e.g., Melipona, Euglossa). Either non-Andean or Andean Merianieae were highlighted in morphospaces, smaller white circles represent ancestral nodes.

Andean Merianieae occupied distinct areas of climatic niche and trait space and generally showed higher disparity than non-Andean Merianieae (Fig. 3, Supplementary Table S6–S8). In phylo-niche spaces, the first three axes explained 80.8% of climatic niche, 74.8% of vegetative, and 54% of floral trait variation. Andean Merianieae are associated with higher elevation and cloud-cover and lower temperatures in climatic niche space (positive PC1, Fig. 3a), and Antillean and Atlantic Forest Merianieae clustered under more seasonal climatic conditions (positive PC2, Fig. 3a). Amazonian and Guiana shield clades (*Adelobotrys* & *Adelbertia*, *Macrocentrum*, *Salpinga*) associated with higher temperatures, higher precipitation and higher productivity. The geographically widespread genus *Graffenrieda* scattered widely across the whole climatic niche space (high disparity, Fig. 3a), whereas *Macrocentrum* showed the smallest climatic niche disparity. Patterns in vegetative trait space did not follow climatic niche space, and Andean Merianieae shared areas of trait space with lowland *Adelobotrys* & *Adelbertia*, *Graffenrieda* and Atlantic Forest Merianieae, and high disparity (Fig. 3b). Species in these clades are shrubs, treelets or trees with moderately sized to large leaves (61 cm² mean leaf area, 385 cm² maximum leaf area) of varying thickness. *Macrocentrum* and *Salpinga*, with their herbal growth form and small, membranaceous/chartacerous leaves (14.4 cm² mean leaf area, 60 cm² maximum leaf area) occupied distinct areas of vegetative space (Fig. 3b). As in climatic space, Andean Merianieae differentiated significantly in floral trait space and showed highest disparity (Fig. 3c, d), with species that have shifted from bee to vertebrate pollination occupying distinct areas of trait space along PC axis 2 (Fig. 3c). These vertebrate-pollination areas in trait space reflect changes in pollinator rewards (nectar or food bodies vs. pollen), modifications of stamens to enable pollen release (nonvibratile pollen release vs. buzz pollination) and corolla shape (pseudo-campanulate vs. open; see Dellinger et al. (2019a) for a detailed description of floral trait space). Bee-pollinated Andean Merianieae, on the other hand, overlapped with non-Andean bee-pollinated species from other clades (except *Macrocentrum* and *Graffenrieda*) along PC1/PC2 (Fig. 3c). They occupied a distinct area of trait space along PC2/PC3 (Fig. 3d), however, reflecting a change to large (>5cm in diameter), sturdy, pollen-rewarding flowers with spread-out stamens adapted to large montane bees, and contrasting with the smaller, delicate flowers of non-Andean bee-pollinated Merianieae. Disparity in the non-Andean clades was significantly lower (Supplementary Table S8).

**Figure 4.**
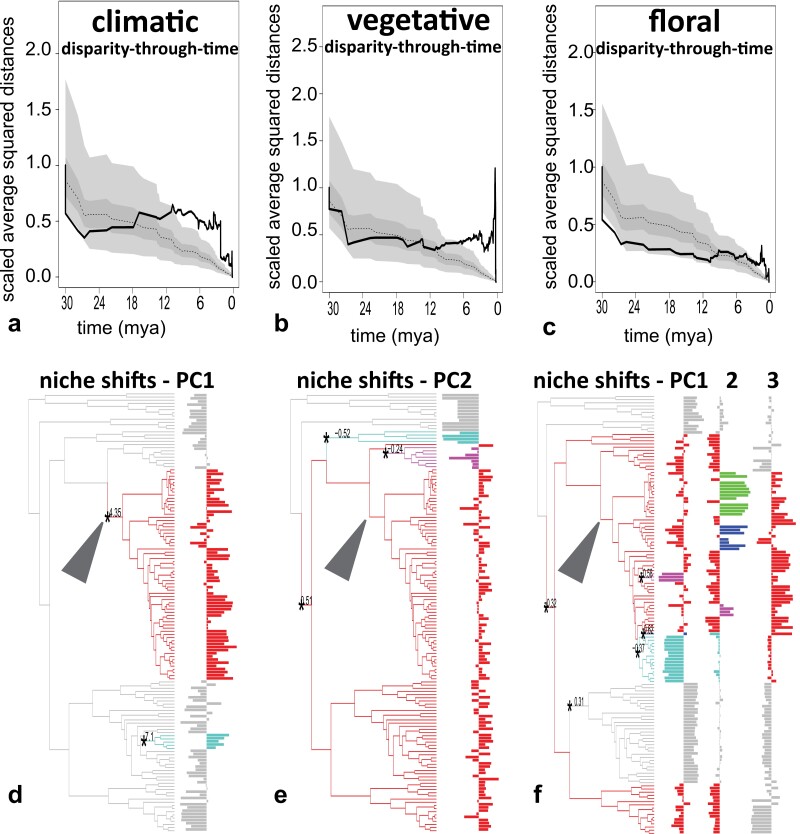
Temporally staggered disparification of climatic, vegetative and floral niches of Merianieae (a-c) and major niche shifts in response to changes in selection regimes (d–f). a–c) Climatic disparity-through-time increases before (18 mya) vegetative (12 mya) and floral (6 mya) disparity-through-time, indicated by the solid line crossing the median (dotted) and 95% confidence intervals (dark gray) for simulated disparification under Brownian Motion. Note that DTT towards the present is likely overestimated here compared with a DTT analysis using a phylogeny with a sampling fraction of 1, where comparatively lower divergence between true sister species is expected; this does not impact the strong temporal staggering in DTT among niche/trait spaces. For detailed patterns in single clades see Supplementary Table S9, Supplementary Fig. S10. d–f) Shifts in niche optima according to OU models (only PC axes with major shifts shown), with d) a major shift to montane climatic conditions on PC1 in Andean Merianieae and montane Graffenrieda, e) a shift in growth form on PC2 from herbs to lianas, shrubs and trees (red, also indicated by *) in Adelobotrys & Adelbertia, Graffenrieda and Andean Merianieae, with Salpinga (turquoise) and Atlantic Forest Merianieae (purple) shifting to distinct optima more similar to herbs (Macrocentrum), and f) a dominant shift to medium-sized (Adelobotrys & Adelbertia, Salpinga) and large (core Merianieae) flowers (red, also indicated by * on node leading to these clades) with distinct niche optima for species that have shifted to vertebrate pollination (passerine: purple, turquoise on PC1, mixed-vertebrate: green, blue, purple on PC2) and a distinct optimum for all Andean Merianieae along PC3. A colour version of this figure appears in the online version of this article.

Exploring the temporal build-up of disparity, we found that disparification in climatic niche space occurred before comparable disparification in vegetative and floral trait space (Fig. 4). Disparity-through-time in climatic niche space increased approximately 18 mya (Fig. 4a), although such clear increases occurred only ca. 12 mya in vegetative, and 6 mya in floral trait space (Fig. 4b, c). All these increases in disparity were not significantly different from simulated DTT under Brownian motion using the MDI statistic (Supplementary Table S9, S10). The estimated increase in disparity in climatic niches 18 mya occurred before most Merianieae clades originated, resulting in homogeneous or decreasing DTT towards the present within clades (most variation among subclades, Supplementary Fig. S10). This onset of climatic niche disparification slightly precedes the global warming during the mid-Miocene climatic optimum, followed by continued disparification during the well-documented global cooling trend since then (Miller et al. 2020). We want to caution that the DTT results stem from present-day climatic data only, and warmer climatic niches were likely available in the Merianieae range in the past than in the present. Given that the cooling since the mid-Miocene occurred at a global scale, however, we can assume that all Merianieae were affected by this event across their distribution range and that the longer period of climatic niche disparification detected in our analyses points towards an important (early) role of climatic niche evolution in general. With the more recent disparification of vegetative and floral traits, our clade-level analyses showed more marked differences in DTT in vegetative and floral trait spaces. *Adelobotrys* and *Adelbertia*, *Macrocentrum* 2&3 and *Salpinga* showed increases in DTT towards the present, indicating increased niche sharing (Supplementary Fig. S10). The other clades also showed increases in DTT within the last 12 million years, but with decreases in DTT within the past 3.5 million years (subclades evolved into distinct niches). In floral niche space, all clades showed an increase in disparification 12 to 7.5 mya, with decreases in DTT within the last 3.5 million years in *Adelobotrys* & *Adelbertia*, Andean and Atlantic Forest Merianieae and *Macrocentrum* 2&3 (Supplementary Fig. S10). These results were consistent also using the phylogenetic hypothesis of Dellinger et al. (2019b) (Supplementary Fig. S17).

Testing whether Merianieae clades have evolved into distinct abiotic niche and biotic trait optima using OU-models, we found high support for shifts and subsequent convergence into distinct optima across spaces (Fig. 4d–f, Supplementary Table S11). In climatic space, we detected two major shifts towards colder montane climatic optima only along PC1 (Andean Merianieae, small group of Andean *Graffenrieda; σ*² 55.6, *α* 7.5; Fig. 4d). Conversely, in vegetative niche space, evolution into three distinct niche optima occurred only on PC2 (mostly summarizing growth form and leaf thickness), with a shift to woody habit (in red) on the branch separating *Macrocentrum* (herbs with membranaceous leaves) from all other Merianieae, a subsequent shift back to herbaceous habit (turquoise) in *Salpinga,* and a shift (purple) to larger trees in Atlantic Forest Merianieae (*σ*² 0.61, *α* 20.92; Fig. 4e, Supplementary Table S11). Finally, shifts in floral trait space reflected pollinator-mediated selection, with five shifts in trait optima detected along PC1. One major shift to larger, zygomorphic flowers occurred along the branch to *Salpinga* and core Merianieae and *Adelobotrys* and *Adelbertia* (Fig. 4f, red), with *Graffenrieda* converging back into the small-flowered optimum of *Macrocentrum* (Fig. 4f, gray). Within Andean Merianieae, passerine-pollinated species showed distinct optima (Fig. 4f, purple, turquoise). Trait shifts associated with pollinator shifts were most clearly reflected along PC2, where we detected a total of 10 shifts, five of which represent separate optima for mixed-vertebrate pollination (nectar-rewarding flowers with pseudo-campanulate corollas; green, blue, purple in Fig. 4f) and passerine pollination (food-body-rewarding flowers with explosive pollen release; turquoise in Fig. 4f; the other five (red) representing convergences in the same bee-pollination optimum as along PC1). Finally, along PC3, we found one major optimum shift encompassing all Andean Merianieae, and corresponding to the overall increase in flower size among Andean Merianieae (Fig. 4f). When comparing the three hypotheses on distinct optima for i) Andean, ii) core, or iii) pollinator-shifted Merianieae, we found highest support across spaces for a single shift in niche optima for Andean Merianieae (6/9 comparisons, Supplementary Table S11). These results were congruent across the phylogenetic hypothesis of Dellinger et al. (2019b) (Supplementary Table S11, Supplementary Fig. S18).

### Effects of Biogeography, Niche, and Trait Evolution on Diversification

Rates of niche/trait evolution differed among clades (Supplementary Fig. S11), with significantly higher evolutionary rates in all niche aspects in Andean Merianieae and *Adelobotrys* and *Adelbertia* than expected by chance (Supplementary Table S12). *Graffenrieda* had higher rates of climatic niche and vegetative than floral trait evolution. In the remaining clades, evolutionary rates did not differ from random expectations, except for a significantly slower rate of vegetative trait evolution in *Macrocentrum* 1 (Supplementary Fig. S11, Supplementary Table S12). We recovered the same qualitative patterns, with significantly faster rates of niche evolution for Andean Merianieae also on the phylogenetic hypothesis of Dellinger et al. (2019b) (Supplementary Table S12).

**Figure 5.**
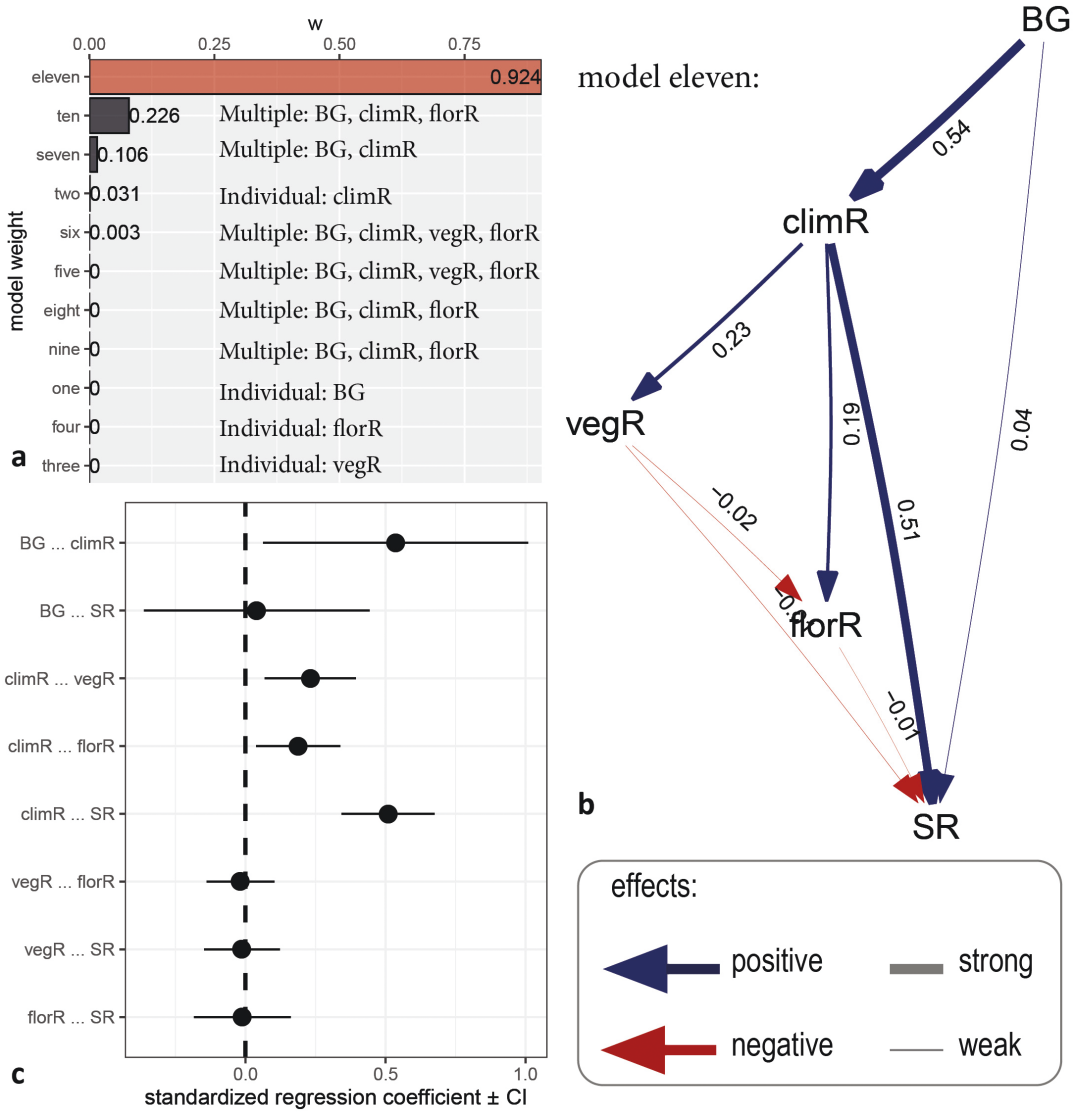
Fit of the 11 path-analytical models and best-fit model (eleven). a) Model eleven, including multiple direct and indirect effects of abiotic and biotic factors resulted as best fit, and was more than two delta CIC better than the next-best-fitting models ten and seven. None of the models with individual effects on speciation rates (one to four) had a good fit to the data (*P* < 0.05). b) In model eleven, biogeographic background (BG) positively impacted rates of climatic niche evolution (climR), which in turn drove speciation rates and also positively affected rates of vegetative and floral trait evolution. The latter did have weak negative effects on speciation rates, whereas biogeographic background itself had a negligible positive effect on speciation rates. c) Standardized regression coefficients of model eleven, with confidence intervals.

Comparing eleven models on individual or multiple direct and indirect effects of abiotic and biotic factors on diversification/speciation rates (Supplementary Fig. S12), we found highest support for the same multieffect model across analyses (model eleven, Fig. 5, Supplementary Table S13). In this best-fit model, biogeographic background (Andean/extra-Andean) drives climatic niche evolution, which, in turn, drives speciation rates (Fig. 5). Climatic niche evolution also drives vegetative and floral trait evolution, but both trait rates only have negligible negative effects on speciation rates. Models with singular direct effects of abiotic/biotic factors on speciation rates never fitted the data well (Fig. 5, Supplementary Table S13). These results were consistent when using rates estimated through DR and BAMM, and the phylogenetic hypothesis of Dellinger et al. (2019b) (Supplementary Table S13, Supplementary Fig. S14, S15).

## Discussion

Combining biogeographic assessments with modeling of Andean uplift, climatic, vegetative, and pollination niche evolution, we here showcase how rapid exploitation of novel ecological opportunities through staggered evolutionary processes explain diversification dynamics. Our findings underscore the importance of evaluating the effects of multiple abiotic and biotic factors, as well as their reciprocity, if we aim to holistically understand the diversification history of a clade. Below, we discuss our approach as well as the complex relationships of biogeography and niche/trait evolution as drivers of diversification in the context of results from other Neotropical lineages.

### The Multiple Direct and Indirect Drivers of Diversification in Merianieae

Although diversification rates increased through time across Merianieae, only the clade that colonized the Northern Andes during a period of major mountain building (and hence formation of novel niche space) 12 mya underwent a major radiation. Our path analyses clearly identified this biogeographic background as main driver of climatic niche evolution, which, in turn, drove vegetative and floral trait evolution, as well as speciation rates. The fact that Merianieae lineages in other montane areas did not undergo comparable radiations emphasizes the importance of timing and adaptive trait evolution (i.e., to altered montane pollinator communities) and refutes the idea that mountain habitat itself drove diversification (Hughes and Atchison 2015; Rahbek et al. 2019; Guedes et al. 2021). In fact, the niche and trait evolution we demonstrate in Andean Merianieae parallels other studies reporting intertwined effects of mountain uplift and adaptive trait evolution across animals and plants (Hughes and Atchison 2015; Liu et al. 2020; Burrez and Muñoz 2021; Figueiredo et al. 2022).

Given the broad recognition of Andean uplift as paramount, continent-wide driver of neotropical diversity (Hoorn et al. 2010; Hughes & Atchison 2015; Bedoya et al. 2021; Figueiredo et al. 2022), the lack of diversification rate shifts also in non-Andean South American clades merits attention. The biogeographic areas we assessed (e.g., Amazon lowlands, Chocó) are characterized by major geomorphological changes since the Miocene, high habitat heterogeneity, and/or marked fluctuations in the distribution of habitat types during Pleistocene glaciation cycles (Willis and Bhagwat 2009; Guayasamin et al. 2021). Regardless of these fluctuating ecological opportunities, our BAMM analyses suggest that species diversity in these areas aggregated continuously towards the present. This is in line with a recent analysis of 150 neotropical plant and animal clades, identifying continuous diversification as the most common, continent-wide process (Meseguer et al. 2022). Among clades showing increased diversification through time, exponential diversification was more commonly observed for plants than animals, and, like in Merianieae, correlated with Andean uplift (Meseguer et al. 2022). Taken together, the diversification process in Merianieae mirrors classic patterns of Neotropical diversification through a combination of continuous diversification in extra-Andean areas, and exponential diversification in the Northern Andes (Rull 2011; Vieu et al. 2022).

Our assessment of niche and trait evolution revealed that Merianieae clades diversified along different axes of abiotic and biotic niche space (Fig. 3), with Andean Merianieae occupying distinct climatic and pollination niche optima. These results parallel findings from other adaptive radiations, underscoring the combined effects of colonization of new areas and adaptive abiotic and biotic niche evolution (Lagomarsino et al. 2016; Barrabé et al. 2019; Lin et al. 2021). Although there is general support for either time-lags (Ackerly et al. 2006; Nürk et al. 2015; Folk et al. 2019) or synchrony of lineage and niche/trait diversification in other systems (Cooney et al. 2016; Castro-Insua et al. 2018; Lapiedra et al. 2021), we found a combination of synchrony (niche evolution) and time lags (trait evolution) with diversification (Figs. 3 and 4). Specifically, disparity-through-time analyses suggested that climatic niche disparification occurred first (starting in the early Miocene), and continued during the subsequent temperature increase in the mid-Miocene climatic optimum and later cooling in the mid-Miocene climatic transition (Miller et al. 2020). Despite the cooling trend since the mid-Miocene being of global impact, only Andean Merianieae radiated, in temporal synchrony with the mid-Miocene pulse of North-Andean uplift. Thus, although results of climatic niche disparity in the past (estimated from present niche data) have to be taken with care, we believe that our results are meaningful in pointing towards an important early role of climatic niche evolution in conjunction with the colonization of newly available montane habitats. These processes were likely followed by disparification of vegetative traits and finally floral syndromes. Our findings are in line with a large body of work identifying abiotic climatic processes as important early determinants of diversification dynamics (i.e., colonization of new biomes), with biotic interactions becoming increasingly important at later stages (Aguilé et al. 2018; Rull 2020; Lapiedra et al. 2021).

The climatic niche disparification in Merianiae was preceded by a single shift in vegetative and floral phenotypic optima (Fig. 4). The vegetative shift encompasses a transition from herbal growth forms with chartaceous leaves characteristic of the Merianieae clades found in the Guiana shield, to woody (shrubs, trees, lianas) growth forms with (sub-)coriaceous leaves in all other Merianieae. This shift in growth form did neither lead to an immediate increase in diversification rates, nor to a burst in vegetative disparity (Fig. 4). Instead, vegetative disparity seems to have increased within clades at later stages of the diversification process (Fig. 3). Similarly, the shift in floral phenotypic optimum along the same branch as the vegetative niche shift, resulting in bigger flowers overall, did not trigger changes in diversification rates or disparification. This early shift in floral phenotypic optimum in clades *Adelobotrys* and *Adelbertia*, *Salpinga*, and all core Merianieae, enabling these clades to exploit medium-sized bees as pollinators (e.g., *Melipona*, *Euglossa*, Dellinger et al. 2022; see Kay and Grossenbacher 2022 for similar pattern in *Costus*), may have acted as important preadaptation for later Andean colonization. The second shift in floral phenotype (increase in flower size) occurred upon Andean colonization, where Merianieae encountered a bee pollinator community made up of bigger, mountain-adapted bees (i.e., *Bombus*, *Xylocopa*, *Centris*). The lack of a comparable increase in flower size despite occurrence in montane areas (Guiana shield, Andes) in the clades *Macrocentrum* and *Graffenrieda* may further explain why these clades never radiated comparably.

The lack of additional vegetative niche shifts in Andean Merianieae was surprising in light of the generally strong associations between the abiotic environment and vegetative traits (Kambach et al. 2023). Across plant lineages, strong links between changing climatic conditions along elevational gradients and vegetative and physiological traits have been identified (Kandlikar et al. 2018; Peng et al. 2020; Homeier et al. 2021). In recent assessments of Andean forests, for example, a reduction in Specific-Leaf-Area and foliar Ca, and an increase in leaf thickness have been reported (Homeier et al. 2021). Although the traits assessed by us (leaf area, thickness, growth form) did not reveal such patterns, we cannot rule out that including more refined physiological measurements might reveal subtler adaptations to the different environments inhabited by Merianieae.

Given extensive empirical and macroevolutionary research on Merianieae pollination (Dellinger et al. 2014, 2019a,b, 2021), we may more readily interpret patterns of floral disparification (Fig. 3). Our results showed that shifts from bee to vertebrate pollinators (and associated floral disparification) were not a prerequisite for Andean colonization, but only occurred within the Andean habitats after colonization. This finding is important in that it supports budding theoretical and empirical work suggesting that the abiotic environment plays a critical role in macroevolutionary pollinator shifts (Thomson and Wilson 2008; Hamilton and Wessinger 2022; Dellinger et al. 2023). In theory, abiotic conditions such as cool, rainy, windy mountain climates, which are highly unfavorable for one pollinator group (i.e., exothermic bees) but not another (i.e., endothermic vertebrates), may significantly reduce flower visitation of the more impacted pollinator group, thereby favoring transitions to the less impacted pollinator group, vertebrates in our case (Dellinger et al. 2021). Since ca. 50% of Andean Merianieae have retained bee pollination, however, this pollination strategy is clearly also successful in the mountain environment. Other processes, such as increased competition for the depauperate montane bee pollinator fauna, reinforced by the rapid increase in species numbers in Andean Merianieae since the mid-Miocene, may have, in addition to impacts by the environment, triggered shifts to vertebrate pollinators. Community-level assessments will help to test this hypothesis (i.e., see Muchhala et al. 2014; Skeels et al. 2021).

### Graphical Models as Synthesis of Diversification Scenarios

The story of Merianieae evolution showcases the value of incorporating multiple factors into assessments of lineage diversification. The need for such inclusive, multifactor diversification assessments has been recognized broadly across the scientific community (Condamine et al. 2018; Uyeda et al. 2018), yet, the implementation of satisfying approaches has been slow. Authors have, to date, mostly used separate models to assess the effects of either an abiotic or biotic factor on diversification (i.e., through state-dependent speciation and extinction (SSE) models, Helmstetter et al. 2023), and even when incorporating both into a study, they were usually retained in separate modeling approaches (Lagomarsino et al. 2016; Condamine et al. 2018; Testo et al. 2019). Likelihood frameworks for the comparison of some models (i.e., time- and environment-dependent birth-death models, Condamine et al. 2018), and promising approaches for modeling the reciprocal effects of continuous (i.e., climatic niche) and discrete (i.e., fruit type) character evolution (Boyko et al. 2023; Tribble et al. 2023) have been proposed. However, results from these models, like our individual analyses on biogeography, paleo-environment-dependent diversification and climatic niche/trait evolution (Supplementary Fig. S1), have mostly remained separate, and their relative impact on each other, and on diversification, have not been incorporated into a single framework.

We here showed how graphical models like phylogenetic path analyses may provide a powerful, hypothesis-driven tool for evaluating the likelihood of reciprocal linkage among drivers of diversification (previously proposed by Höhna et al. 2014; Uyeda et al. 2018). In contrast to data-driven approaches (i.e., BAMM, OU models without apriori definition of shift regimes), graphical models require the apriori formulation of specific, causal hypotheses (Fig. 1, Supplementary Fig. S12). Although additional follow-up studies are needed to dissect specific mechanisms (e.g., role of climate variation in leaf trait evolution), graphical models allow us to detect significant interactions and test competing causal paths based on comparative, macroevolutionary data (von Hardenberg and Gonzalez-Voyer 2013; Uyeda et al. 2018). In the case of Merianeae, a model linking biogeography to diversification via climatic variation suggests that research into speciation along climatic gradients (e.g., elevation) will be the most informative for understanding mechanisms underlying diversification at finer scales.

In our approach, we derived tip rates from our initial (separate) analyses as variables to determine the most likely hypothesis on drivers of diversification, thereby using PPA as a synthesis across our results (Supplementary Fig. S1). Using tip rates as meaningful estimates of past evolutionary processes was possible because major historic changes in tip rates persist until the present in Merianieae (Figs. 2 and 4). This simple approach might be challenging in systems with pronounced rate variation through time, and might require the use of more sophisticated measures (i.e., including rate estimates at nodes). Further, our dataset has some limitations regarding sampling fraction (46%), clade size (139 spp. sampled), and time calibration (few fossils), that are, unfortunately, inherent to many evolutionary studies. Low sampling fractions, small clade sizes, and uncertainty around divergence time estimates may all increase the likelihood of detecting significant effects of single factors on diversification dynamics (Helmstetter et al. 2023), and this might hold equally true when assessing the effects of multiple factors. To evaluate whether these data-inherent features also impact our results, we have 1) randomly subsampled our dataset to 50% (70 spp., reducing the sampling fraction to ca. 23%) and 2) run our analyses across two different phylogenetic hypotheses (Dellinger et al. 2019b; Reginato et al. 2022) which differ in calibration techniques and age estimates. Across analyses, we recovered the same mid-Miocene burst in diversification among core/Andean Merianieae, conferring robustness to our results. Our results are also robust when delineating biogeographic areas differently (using an ecoregion rather than geomorphological delimitation, Morrone et al. 2022), showing that the Merianieae radiation is confined to the Andean bioregion. This consistency in our results is promising in that it suggests that when a clade’s phylogeny, distribution range, and niche/trait space are sampled evenly (like in Merianieae), we might indeed detect real macroevolutionary patterns.

### Conclusions

Here, we show how quantifying the relative effects and timing of abiotic (biogeography, climatic niche evolution) and biotic (vegetative and floral traits) factors on diversification dynamics within a single analytical framework gives a nuanced, balanced perspective on the interlinked processes underlying macroevolution. The path-analytical approach proposed by us is versatile in that it allows for the formulation and testing of explicit hypotheses tailored towards different study systems. We hope that our approach will serve as a blue-print for studies on the intertwined drivers of diversification in other clades in the future, and will, on the long run, allow for a joint synthesis on the relative importance and timing of abiotic niche and biotic trait evolution in diversification processes.

## Supplementary Material

Data available from the Dryad Digital Repository: https://doi.org/10.5061/dryad.bvq83bkdx

## Funding

This work was supported by Austrian Science Fund FWF grant T-1186 to ASD, National Science Foundation NSF award 2055525 to LPL and NSF DEB 1553114 to SDS.

## Data Availability

Data available from the Dryad Digital Repository: https://doi.org/10.5061/dryad.bvq83bkdx.
